# Discriminating Formal Representations of Risk in Anterior Cingulate Cortex and Inferior Frontal Gyrus

**DOI:** 10.3389/fnins.2018.00553

**Published:** 2018-08-14

**Authors:** Rena Fukunaga, John R. Purcell, Joshua W. Brown

**Affiliations:** Department of Psychological and Brain Sciences, Indiana University Bloomington, Bloomington, IN, United States

**Keywords:** risk signal, decision making, anterior cingulate cortex, inferior frontal gyrus, Gambling

## Abstract

Considerable debate persists around the definition of risk. Depending on the area of study, the concept of risk may be defined as the variance of the possible outcomes, the probability of a loss, or a combination of the loss probability and its maximum possible loss. Mounting evidence suggests the anterior cingulate cortex (ACC), including the surrounding medial prefrontal cortex (mPFC), and the anterior insula/inferior frontal gyrus (IFG) are key neural regions that represent perceived risks. Yet it remains unclear which of these formalisms best accounts for the pattern of activation in brain regions representing risk, and it is also difficult to disentangle risk from value, as both contribute to perceived utility. To adjudicate among the possible definitions, we used fMRI with a novel gambling task that orthogonalized the variance, loss probability, and maximum possible loss among the risky options, while maintaining a constant expected value across all monetary gambles to isolate the impact of risk rather than value. Here we show that when expected value is controlled for ACC and IFG activation reflect variance, but neither loss probability nor maximum possible loss. Across subjects, variance-related activation within the ACC correlates indirectly with risk aversion. Our results highlight the variance of the prospective outcomes as a formal representation of risk that is reflected both in brain activity and behavior, thus suggestive of a stronger link among formal economic theories of financial risk, naturalistic risk taking, and neural representations of risk.

## Introduction

Cognitive neuroscience studies have used risky decision-making tasks to identify a network of brain regions involved in predicting and evaluating the potential outcomes of an action, which often influences decisions to avoid anticipated risks (Preuschoff et al., 2008; Jahn et al., 2011; Barkley-Levenson et al., 2013; van Duijvenvoorde et al., 2015). Across such studies, however, are sizeable differences in the treatment of risk, as risk constructs often represent various meanings across a wide range of disciplines.

In its most basic form, risk involves increasing uncertainty about an outcome and possible undesirable consequences. Earlier work suggested that greater risk involves greater variance or heavier tails of a distribution (Rothschild and Stiglitz, 1970). Currently the concept of risk generally equates to variance in the areas of economics and finance (Markowitz, 1952; Slovic and Lichtenstein, 1968). This definition of risk, however, sometimes misses the broader concept of risk as entailing aversive consequences. For example, an equal probability of gaining either $1 million or $2 million would entail a large variance in the outcome, although neither outcome would necessarily be considered aversive. In other disciplines, risk has different meanings that focus specifically on the possibility of aversive outcomes rather than simply variance. In health psychology for example, risk involves the probability of a bad outcome such as getting a disease (Rothman and Salovey, 1997), and in other contexts, risk analysis involves both the probability of a particular aversive outcome and the severity of the aversive outcome (Saaty, 1987).

Functional MRI studies have suggested a variety of often competing theories of how risk is represented neurally, to the point that a direct test of competing neural risk constructs is needed. Earlier studies of risk using a card guessing task found that dorsal anterior cingulate cortex (dACC) is activated by greater “uncertainty” regarding an outcome (Critchley et al., 2001). Subsequent studies with a similar task treated risk as identical to variance and found strong representations of risk as variance, which were distinct from reward representations (Preuschoff et al., 2006). This distinction has been challenged by others who argue that appetitive and aversive factors such as reward and risk are represented by a scalar composite signal of value, without a separate neural representation that is activated specifically by aversive properties of a gamble (Tom et al., 2007). A related study found heightened dACC activity with an increased “spread of outcomes” or greater variance, but did not covary with risk attitudes – although the IFG activity did covary with risk aversion (Christopoulos et al., 2009). A set of follow-up studies showed that neural representations of variance were distinct from variance prediction errors in the bilateral insula (Preuschoff et al., 2008), and that such signals are stronger in those who tend to avoid risk (Rudorf et al., 2012). Previous work has also implicated the role of cortical regions such as the prefrontal cortex and parietal lobe, in addition to the insula, during decision-making to minimize loss, maximize gain, and assess gain probability (Venkatraman et al., 2009).

The same insula or inferior frontal gyrus (IFG; Amiez et al., 2016) and cingulate regions found to represent variance have also been proposed to represent alternative risk constructs such as loss probability (Brown and Braver, 2005), the magnitude of a loss (Brown and Braver, 2007; Canessa et al., 2013), and the related construct of loss aversion (Mohr et al., 2010; Fukunaga et al., 2012). In one study, the authors went so far as to propose that there is no formal explicit representation of risk in the brain, but only an implicit representation of risk seen as a reduction in value-related brain activity (Tom et al., 2007). As a result of this lack of clarity, a systematic decomposition and study of various risk concepts has been called for in the literature (Venkatraman et al., 2009; Schonberg et al., 2011) to resolve discrepancies across disciplines and build a bridge between the literatures on economic risk taking and more naturalistic risk taking (Schonberg et al., 2012).

**FIGURE 1:**
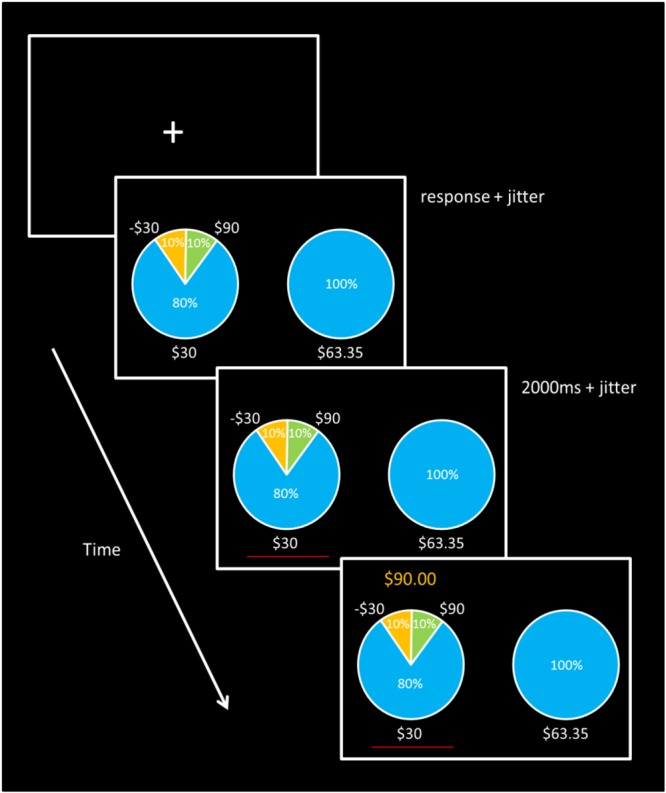
Task Design. Trials began with a blank screen and fixation cross lasting 1.5, 3.5, 5.5, or 7.5 s. Each gamble was then presented with its corresponding ST option. Participants were give unlimited time to make a response, after which a red line appeared for at least 2 s under the selected option, followed by the gamble outcome displayed above the chosen gamble for 1.5 s. Sure thing outcome payment amounts were adjusted for each gamble to maintain 50% gamble choice probability for each of the five different types of gambles.

Here we aim to answer this call and directly test competing theories about how risk is represented at the neural level. This fMRI study examines the underlying neural correlates of risky decision-making by directly disentangling brain signals associated with three commonly applied definitions of risk: variance, loss probability, and the magnitude of potential loss, while controlling for expected value. A 2-arm bandit task (see **Figure 1**) was designed to orthogonalize the three ‘risk’ conditions, while maintaining a constant expected value across all gambles. Participants chose between a gamble and a “sure thing (ST)” outcome, with the sure option adjusted dynamically to the certainty equivalent (CE) for each gamble in order to ensure that individuals chose the gamble over the ST on 50% of trials. There are potentially other factors that we could not decorrelate in the present design, including the skewness of the gamble, the entropy of the gamble, and the maximum possible win. Still, to our knowledge, this is the first neuroimaging study to implement an experimental paradigm that allows for findings to discriminate which among a set of risk formalisms best accounts for brain activity at the time of risky decision-making involving monetary gain and loss outcomes, and how such activity may correlate with avoidance behavior.

## Materials and Methods

### Participants

A total of 25 participants (14 females), ages 19–30 years (*M* = 24.24 years, *SD* = 3.00) completed the task paradigm. Thirty-two participants were initially recruited from the student body of Indiana University, Bloomington. All participants provided written informed consent and were right-handed (with the exception of one participant), and met standard health and safety requirements. Participants were paid $25/h for participation, plus performance bonuses (*M* = $4.51, range = $3.69–$5.76) based on points earned during the experimental task. Subjects were told “…you will also receive a bonus amount between $0 and up to $30. How much money you receive will depend on your [task] winnings…” (See **Supplemental Materials** for full script and bonus calculation). Losses incurred were only taken out of the subject’s possible task bonus, not from the hourly pay rate. There is evidence that giving feedback about the outcome of a gamble alters behavior (Jessup et al., 2008), and furthermore that giving abstract “points” as reward (and later converted to money) serves as an incentivizing reinforcer (Weiner, 1971; for review, see Hackenberg, 2009). All procedures were approved by the Indiana University Bloomington Institutional Review Board.

Our final analysis was conducted on 25 subjects, excluding a total of 3 participants for unusable data (severe head movement, *n* = 1; non-specific abnormal radiological findings, *n* = 1; incomplete data collection due to technical malfunction, *n* = 1), and 4 participants who discontinued scanning (due to self-reported claustrophobia, *n* = 1; physical discomfort, *n* = 1; severe anxiety, *n* = 1; and concentration problems, *n* = 1).

### Novel Gambling Task Procedure

The task procedure is depicted in **Figure 1**. On each trial, participants were presented with a choice between a gamble (option 1) or a ST (option 2). The ST option consisted of a specified payoff with 100% certainty. Each option appeared in the form of a pie chart, and the payoff distributions were explicitly revealed to the participants. The options were shown counterbalanced on either the left or right side of the computer screen. There were five different gambles, each with three possible outcome values. The properties of the gamble options –probability of loss [P(*Loss*)], maximum possible loss [Max(*Loss*)], and variance (*Var*)– were constructed to be mutually uncorrelated. We did this by first creating four gambles that differed from each other in terms of the various properties of risk, while maintaining a constant expected value ($30) across all five gamble options. Despite this, the risk properties of variance, maximum possible loss, and loss probability were still correlated with each other across the gambles. Thus, we added the fifth gamble with parameters optimized to break the correlation between pairs of formal properties P(*Loss*), Max(*Loss*), and (*Var*), (Christopoulos et al., 2009), so that the pairwise correlations are all essentially zero. In other words, the fifth gamble served to orthogonalize the constructs across the gambles. It was also necessary for each gamble to include at least three possible outcomes in order to decorrelate the formal properties of the gambles. The correlation coefficients (*R*^2^) were all < = 0.001 pairwise among the three formalisms. Applying the pairwise approach ensured that any brain activity in the fMRI general linear model (GLM) that loaded on one of the constructs would not be confounded with or shared with the other constructs. The derived (risk) properties for each stimulus conditions are shown in **Table 1**.

**Table 1:** Task parameters for risk formalisms.

	Gamble 1	Gamble 2	Gamble 3	Gamble 4	Gamble 5
EV	30	30	30	30	30.01
P(*Loss*)	0.1	0.1	0.25	0.25	0.13
Max(*Loss*)	-30	-65	-8	-30	-23
*Var*	240	601.66	240	600	6982.72
Skewness	0	0	0	0	12460227.5
Entropy	0.92	0.92	1.5	1.5	0.83

*Gamble 1 = Baseline, Gamble 2 = Control Probability of Loss, Gamble 3 = Control Variance, Gamble 4 = Control Maximum Possible Loss, Gamble 5 = Orthogonalization Condition. For details of the gamble offers, see **Supplementary Table S1**.*

Each trial began with one of the five gamble options and presented with its corresponding ST option (100% probability of earning money), with the gamble appearing on the left in 50% of trials to avoid a potential confound between motor response and choice of gamble vs. ST. Participants were given no time limit to indicate a choice, but were trained behaviorally on the task outside of the scanner (∼20 trials) to make judgments within a few seconds. Once the participant indicated a left or right choice with a button press by the corresponding left or right index finger, a red line appeared for 2 s (61% of trials), 4 (25%), 6 (10%), or 8 (4%) s, based on an exponential distribution (Dale, 1999), under the selected option to confirm that a choice was recorded. This was followed by the amount of the paid gamble displayed above the chosen gamble for 1.5 s. After receiving feedback, a blank screen, serving as a jittered interval, appeared lasting either 1.5, 3.5, 5.5, or 7.5 s, again based on the same exponential distribution function as following the response (Dale, 1999) prior to the start of a new trial.

To reduce the confounding factors of choice probability (Braver et al., 2001), it is important to ensure that the gamble and ST options are each chosen on 50% of the trials for each of the five gambles (Christopoulos et al., 2009). The reason is that if the probability of one choice is substantially lower than the probability of another choice, then the infrequent choice will likely be associated with greater activity in the medial PFC (Jessup et al., 2010; Alexander and Brown, 2011). This would confound effects we might see in the medial PFC. To this end, a successive approximation method and a linear control algorithm were used to track and estimate the CEs for each of the five corresponding gambles separately. The CE is the ST payoff value that is equally preferred to the gamble alternative. In other words, this is the minimum amount of money offered as a ST payout for which the individual is indifferent between a gamble and the ST. The concept of CE is important for our understanding because it can be applied to label different attitudes toward risk taking. That is (for an uncertain alternative specified in terms of gains), one is considered risk averse if the CE is less than the expected value of the gamble, whereas one is labeled as risk seeking if the CE is greater than the expected gain (Rudorf et al., 2012).

Each participant’s experimental session proceeded as follows, as described in earlier work (Paulus and Frank, 2006). During the first eight trials of each of the five gamble conditions, the ST options are narrowed based on a successive approximation method. The upper and lower bounds are initially set to the maximum and minimum payoffs of the gamble in the corresponding condition. Participants are presented with a ST that is the lower bound plus two-thirds of the difference between the upper and lower bound. If participants choose the ST, then the ST becomes the new upper bound. If participants instead choose the gamble, then the ST becomes the new lower bound. On the next trial with the same condition, the ST is the lower bound plus one-third of the difference between the upper and lower bound. After eight trials for each condition, the initial certainty equivalent (ICE) is then estimated as the average of the upper and lower bounds. This CE procedure was implemented for each participant during the experimental task guided, in part, by prior neuroimaging work that have also used this approach (Paulus and Frank, 2006).

After the initial phase of eight trials per each of the five gambles, the ST that is offered in subsequent trials followed a modified second-order linear control signal (i.e., a modified proportional–integral–derivative or PID controller). The control signal is designed to (1) maintain the choice probability as close to 50% as possible for each condition, and (2) minimize the trial-to-trial fluctuation in the presented ST option, in order to ensure that the decision was not too easy for the participants. To accomplish this, a CE estimate or a “new” ST was computed as follows:

$$\begin{array}{c} \mathrm{CE}\;\leftarrow \;\mathrm{CE}\;+\;30\times [\mathrm{GambleTrackingGain}\times \mathrm{IntegralError}\;+\;\mathrm{GambleSpringTerm}\times (\mathrm{CE}\;-\;\mathrm{ICE})] \end{array}$$

In this equation, the IntegralError represents the integral term, which is the cumulative number of gamble choices minus the cumulative number of ST choices for the condition. This is essentially the degree to which the cumulative gamble choice probability differed from 50%. The GambleTrackingGain is set at a constant 0.3, and the Gamble Spring Term at a constant -0.01. Here the Proportional error term with the GambleSpringTerm coefficient used the difference of the current and ICE estimates instead of the difference of the gamble choice probability from 50%. This effectively anchored the current CE estimate to the initial estimate in order to increase stability. The ICE is estimated by the average upper and lower bounds of the CE at the end of the initial phase of the eight trials for each gamble. These controller coefficients were determined by Monte Carlo optimization procedures (before any human subjects were run) with simulations of participant choice behavior, and with the objective of maintaining the subject choice probabilities as close to the indifference point (i.e., 50% gamble choices) as possible. In the end, the algorithm performed well at controlling the choice probabilities, and the 50% choice probability of gambles was maintained for each of the five gamble conditions across the 180 trials (45 trials per block) completed over the course of four runs. We also explored using a linear damping term based on the change in CE per trial, but this did not significantly improve the performance and thus was omitted. The final CE calculated for each gamble for each participant was the average of all ST options presented alongside the corresponding gamble. For behavioral analysis, we examined whether CEs for each participant correlated with the neural representations of variance, loss probability, or maximum possible loss of each gamble, to draw a direct brain-behavior relationship. We will share the experimental script, written in E-prime, with interested parties on request.

### Self-Report Measures

Participants (*n* = 24) completed the Domain Specific Risk-Taking Attitude Scale (Weber et al., 2002), a self-report measure that assesses risk-taking and risk perception within the domains of recreational, social, ethical, health/safety, and financial risk taking. One of the fMRI subjects did not complete the survey. The DOSPERT measures likelihood of risk taking behavior, perceived risk, and expected benefits across each of these four domains. The financial domain consists of gambling and monetary investment subscales, containing questions involving scenarios in which college students may not identify [e.g., “Betting a day’s income at the horse races” (item 19), “Investing 5% of your annual income into speculative stock” (item 15)]. We thus expected that questions about the likelihood of participants engaging in risky behavior would not be sensitive measures since they were unlikely to engage in them regardless of risk perception. Instead, we surmised that the scale’s measurement of perceived risk and expected benefit would better capture risk evaluation.

### fMRI Acquisition and Data Preprocessing

Images were acquired on a 3T Siemens TIM Trio scanner using a 32-channel head coil. Functional BOLD data were collected at a 30° angle from the anterior commissure-posterior commissure line in order to maximize signal-to-noise ratio in the orbital and ventral regions of the brain (Deichmann et al., 2003). Functional T2^∗^ -weighted images were acquired using a gradient echo planar imaging sequence with 35 axial slices and 3.44 mm × 3.44 mm × 3.75 mm voxels (30mm × 3.8 mm interleaved slices; TR = 2000 ms; TE = 25 ms; 64 × 64 voxel matrix; flip angle = 70; field of view = 220mm × 220mm). Four runs of data were collected with 285 functional scans each. High-resolution T1-weighted MPRAGE images were collected for spatial normalization excitation consisting of 192 sagittal slices (256 × 256 × 196 voxel matrix of 1 mm × 1 mm × 1 mm voxels, TR = 1800 ms; TE = 2.67 ms; flip angle = 9) at the end of each session.

Functional data were spike-corrected on a voxel-by-voxel basis to reduce the impact of artifacts using AFNI’s 3dDespike^1^. Subsequent preprocessing was done using SPM5 (Welcome Department of Imaging Neuroscience London, United Kingdom^2^). Functional images were corrected for differences in slice timing using sinc-interpolation (Oppenheim et al., 1999) and head movement using a least-squares approach and a 6-parameter rigid body spatial transformation. Once the resulting images were co-registered to the structural image and normalized to standard Montreal Neurological Institute (MNI) space, the resulting functional images were then spatially smoothed with an 8-mm^3^ full-width-at-half-maximum isotropic Gaussian kernel.

### fMRI Analysis

Functional neuroimaging data were statistically analyzed based on a GLM with random effects implemented in SPM5. Each individual subject’s GLM was estimated with a canonical hemodynamic response function (HRF) with no derivatives, a microtime resolution of 16 time bins per scan, a high-pass filter cutoff of 128 s using a residual forming matrix, autoregressive AR(1) to account for serial correlations, and restricted maximum likelihood (ReML) for model estimation.

The GLM included a total of 17 regressors: two constant terms, 6 motion regressors, and 9 regressors for experimental conditions during the choice period and the feedback period. The nine event regressors were labeled as follows*: Choice* (which modeled all event times in which the subject chose the gamble or ST), *Choice^∗^P(Loss), Choice^∗^Max(Loss), Choice^∗^Var, Gamble, ChoiceDur, Acknowledge, FeedbackWin, FeedbackLoss*. The rationale for these regressors is as follows. First, every event included a Choice, whether it was for the Gamble or the ST. The *Choice* regressor modeled the main effect of a choice event, regardless of whether the choice was for the Gamble or the ST. Next, to explore the nature of the risk-related increase in activation, we included several mean-centered parametrically modulated regressors for the *Choice* event, namely individual regressors that modeled the variance, loss probability, and maximum loss of the chosen option. Each parametrically modulated regressor was mean-centered and normalized (z-scored) by dividing the regressor by its standard deviation, which compensated for the fact that the raw value ranges were different (i.e., larger for variances, and smaller for probabilities). This provided parametric modulators (indicated by *Choice^∗^P(Loss), Choice^∗^Max(Loss), Choice^∗^Var*), aligned to the time of response, to identify brain regions where activation was positively or negatively correlated with the three types of formal risk representations: (1) Loss probability: P(*Loss*)^∗^; (2) Maximum Possible Loss: Max(*Loss*)^∗^; (3) Variance: *Var*^∗^. Next, we considered that there may be a main effect of activation specifically in trials when subjects chose the gamble option (Cohen et al., 2005; Fukui et al., 2005; Rao et al., 2008; Krawitz et al., 2010), hence the inclusion of a regressor specifically to model subjects choice of the gamble option at the time of response. Note that given SPM’s limitations and our desire to include parametrically modulated regressors for all choice trials, we could not separately model trials in which subjects chose the gamble vs. trials when they chose the ST, without creating a degenerate design matrix. An epoch regressor (*ChoiceDur*) with an onset at the time of presentation and spanning the duration of the choice event was also included to capture and control for potentially confounding effects of reaction time (time on task) on regions heavily active during choice behavior (Grinband et al., 2011). Feedback events were modeled by three regressors, one to designate the time when subjects were informed that a choice was recorded (*Acknowledge*), another to model the subsequent feedback events, specifically a loss event (*FeedbackLoss*) and lastly to indicate a win event (*FeedbackWin*). Regions identified as loading significantly on the parametrically modulated Choice regressors of P(*Loss*)^∗^, Max(*Loss*)^∗^, *Var*^∗^ were further analyzed to determine whether their activity might correlate with avoidance of gambles across subjects, i.e., whether brain activity correlated negatively with the CEs in such a way that the BOLD signal would be logically sufficient to serve as avoidance signals with respect to the gamble options.

To examine changes in the neural basis of risk representation in the medial prefrontal region, we also planned an independent region-of-interest (ROI) analysis based on an independent mask taken from a previous study that identified risk signals in the ACC (peak voxel = MNI 6, 26, 24) and bilateral IFG /anterior insula (peak voxels = MNI –44, 16, –8 and 48, 20, –6) (Fukunaga et al., 2012). We planned independent ROI masks to be applied systematically across the three risk constructs to avoid ‘double dipping’ of the same dataset (Kriegeskorte et al., 2009).

#### Parametrically Modulated Analysis

We used parametric modulation analysis to address the main question of whether distinct brain regions represent any of the three proposed constructs of risk. We focused on three main parametrically modulated regressors modeled at the time of decision-making: (1) Loss probability: P(*Loss*)^∗^, (2) Maximum Loss: Max(*Loss*)^∗^, and (3) Variance: *Var*^∗^. Regions were defined as contiguous voxel clusters passing a cluster defining threshold of *p* < 0.001, unless otherwise specified. Clusters identified in this way were further evaluated for whole-brain significance with SPM5 based on the cluster size, i.e., cluster corrected *p*-values with a cluster corrected alpha = 0.05. As an additional check to control for potentially inflated cluster significance (Eklund et al., 2016), we computed the minimum significant cluster size with a version of AFNI’s 3dClustSim compiled Jan. 11, 2017, using our population brain mask of valid voxels. This analysis suggested that with a cluster defining threshold of *p* < 0.001 and cluster-corrected alpha = 0.05, clusters of size 73 voxels (contiguous voxel faces) or greater are significant. We thus identified as significant clusters that exceeded this size. We will share our anonymized data and analysis scripts with interested parties on request.

## Results

### Behavioral Analyses

**FIGURE 2:**
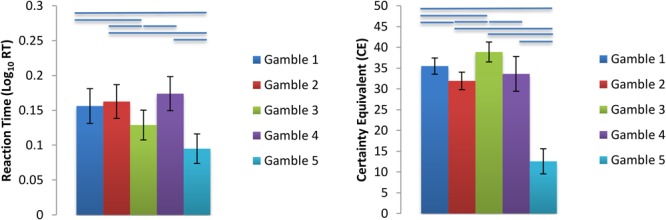
Log_10_ Transformed Mean Reaction Times and CE s for each gamble. Gamble 1 = Baseline, Gamble 2 = Control Probability of Loss, Gamble 3 = Control Variance, Gamble 4 = Control Maximum Possible Loss, Gamble 5 = Orthogonalization Condition. Bars above gamble pairs denote significance at *p* < 0.05. For further statistics see **Supplementary Tables S2–S7** and **Supplementary Figure S3** in the **Supplementary Materials**.

As expected, all subjects chose the gambles and the ST options equally often (gamble chosen average of 50.0% of trials across population; all subject *p* > 0.9, Fisher Exact test). We tested to see whether the *Var*, P(*Loss*), or Max(*Loss*) constructs accounted for behavioral preferences, in particular, the CE values and reaction times for all trials (RT) found for each gamble (**Figure 2**). We fitted six linear mixed effect models with lmerTest in R and computed *p*-values based on degrees of freedom computed with Satterthwaite’s method. All RTs were greater than 350 ms, thus trimming of fast RTs was unnecessary. However, RTs were trimmed if they were 3 SDs above or below the subject’s mean RT, resulting in a total of 64 (1.44%) trials removed due to slow responding. Statistical analyses below were performed on the log_10_ transformed RTs to correct for deviations from normality (see **Supplementary Figure S6** for a histogram of all raw RT data). The six models used log RT or CEs as dependent variables, with model *Var*, P(*Loss*), or *Max*(*Loss*) as fixed effects. All models had random intercepts and slopes grouped by subject as a random effect, and *p*-values reflect two-tailed tests. Greater gamble variance trended toward smaller RTs [*t*(6.13) = -3.49, *p* = 0.13] and smaller CEs [*t*(24.23) = -6.41, *p* = 1.2 × 10^-6^], consistent with risk aversion. Greater gamble P(*Loss*) had no association with RTs [*t*(24.03) = 0.83, *p* = 0.42] but was associated with increasing CE [*t*(24.0) = 5.49, *p* = 1.2 × 10^-5^]. Greater Max(*Loss*) was associated with smaller RTs [*t*(23.93) = -3.72, *p* = 0.001] but had no relationship with CEs [*t*(23.98) = 0.047, *p* = 0.96]. As a control, we tested whether repeated presentations of the same gamble might causes preferences to shift over time, but we found no evidence of this (**Supplementary Material**).

We also conducted a logistic regression in each subject to identify which factors determined the probability of choosing the ST option, and we found that greater ST values and greater gamble variance were associated with a greater probability of choosing the ST, but loss probability and maximum possible loss were not significant predictors of choice probability (**Supplementary Figure S4** and **Supplementary Table S9**).

### fMRI Analyses

#### Loss Probability and Maximum Possible Loss During Choice

Neither the P(*Loss*) nor Max(*Loss*) regressors showed significant effects anywhere in the whole brain analysis.

#### Variance Regressor During Choice

**FIGURE 3:**
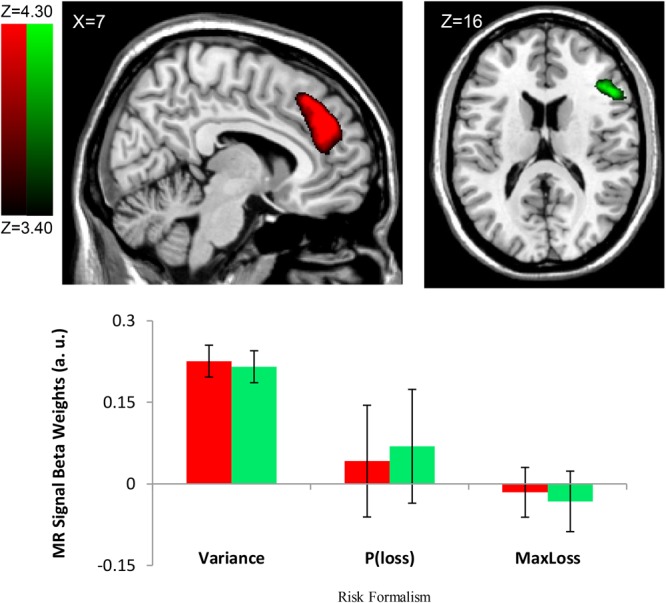
Risk Formalism Regressor Loadings During Choice. Top left: ACC region defined by significant loading on Variance regressor (Var). Top right: Region of IFG/AI also loading on variance regressor. Other regressors were not significant. Bottom: only the variance regressor was significant. No other regions showed significant loading on P(*Loss*) or Max Loss regressors. Note in the bar plot, the Variance regressor loading is significant at the level of whole brain correction. Regions shown here were identified by whole-brain cluster correction. We report no *p*–value for the variance regressor ROI loading specifically because doing so would amount to double-dipping (Vul et al., 2009). ROIs shown at FDR *q* < 0.01.

Consistent with the risk definition from finance theory, we found evidence to support outcome variance as a neural basis of risk representation. The *Var^∗^*Choice (Variance) regressor showed increasing activity (positive correlation; β’*s* > 0) in right Middle Frontal Gyrus/ IFG (cluster size *k* = 2018, peak voxel MNI 42, 18, 46; *p* < 0.002, cluster corrected, two-tailed) (see **Figure 3**) and the right Medial Frontal Gyrus (rMFG) including the ACC (cluster size *k* = 4098, peak voxel: MNI 16, 32, 44; *p* < 0.002, cluster corrected, two-tailed) (see **Figure 3**) as the variance increased for the chosen gamble option. Similar effects were found in additional regions including the right Angular/Supramarginal Gyrus (cluster size k = 1236, peak voxel: MNI 52, -50, 28; *p* < 0.002, cluster corrected, two-tailed).

#### Additional ST Regressor

As can be seen in **Supplementary Figures S1**, **S2**, Gamble 5 had the highest variance and was also associated with the lowest CE s. This raises a question of whether the variance-related activation could reflect lower ST values rather than higher variance of the gamble. Logically, the lower ST values are caused by aversion to the (higher variance) gamble, as subjects prefer even smaller ST values to the gamble. This suggests that greater BOLD signal loading must ultimately reflect the greater variance of the gamble rather than the resulting lower ST values. Nevertheless, to explore this, we reanalyzed the data with a regressor for the ST values included as a fourth parametrically modulated regressor, in addition to the original three modeling variance, loss probability, and maximum possible loss, in a separate set of GLMs. The ST regressor showed no significant loading, suggesting that the ST value of each gamble did not contribute to differences in activation in the ACC or insula, or in any other regions that might be related to reward anticipation of the ST.

Nevertheless, the variance regressor and the ST regressor were negatively correlated, because aversion to higher-variance gambles necessarily results in lower ST offers, making it difficult to decorrelate the two. In order to further analyze these results, the initial eight trails of each condition were analyzed independently, as the ST options are narrowed based on a successive approximation method during these trials, causing the largest amount of variation in the ST options relative to the variance of the gamble. This might in principle make it easier to see BOLD signal loading on the ST regressor, but in practice this was not the case. The first eight trials showed results consistent with the remaining trials. BOLD signals loading on P(*Loss*), MaxLoss, and ST were non-significant, however, the Var regressor yielded a significant cluster in the rMFG (cluster size *k* = 1953, peak voxel MNI 8, 32, 38; *p* < 0.002, cluster corrected, two-tailed) and right MFG/ IFG (cluster size *k* = 166, peak voxel MNI 28, 16, -16; *p* < 0.002, cluster corrected, two-tailed). We note with caution, however, that the order in which the parametrically modulated ST regressor is entered matters – if we enter the ST regressor first to model all trials, then it does load significantly in a region including the ACC (cluster size *k* = 2059, peak voxel MNI -12, 40, 14; *p* < 0.002, cluster corrected, two-tailed). This means that we cannot ultimately rule out greater activation in response to lower ST values, but we note that the lower ST values are themselves a consequence of the greater variance of a gamble, leaving greater variance as the driving factor.

#### Feedback Contrasts

The difference between *FeedbackWin* and *FeedbackLoss* regressors yielded significant clusters in the ACC, insula, and orbitofrontal cortex (see **Supplementary Table S8**). These findings are congruous with previous literature implicating the association of these regions with error detection, feedback integration, and decision value (Hare et al., 2008; Simões-Franklin et al., 2010; Tsuchida et al., 2010). We do not treat these results further here as they are not central to our questions regarding risk representation. For completeness, we note that the *Var^∗^* regressor also showed decreased activity (negative correlation; β’*s* < 0) in the Lingual/Posterior Cingulate Gyrus and bilateral Paracentral Lobule (**Table 2**), although the significance of this is less clear.

**Table 2:** Positive and negative loadings for variance regressor.

			Peak MNI Coordinates				
Region	Laterality	Cluster Size	X	Y	Z	Max stat t	P Cluster Corrected
**Positive Loading**							
Angular/Supramarginal Gyrus	R	1236	52	–50	28	7.46	<0.001
Medial Frontal Gyrus	R	4098	16	32	44	7.17	<0.001
Middle Frontal Gyrus	R	2018	42	18	46	6.75	<0.001
Fusiform Gyrus	L	289	–34	–52	–14	5.52	<0.001
Inferior Parietal Lobule	L	181	–52	–36	40	4.96	0.001
Brodmann 13/Insula	L	91	–32	14	–10	4.81	0.044
Parahippocampal Gyrus	R	102	26	–32	–16	4.8	0.027
Inferior/Middle Temporal Gyrus	R	145	58	–36	–14	4.75	0.005
**Negative Loading**							
Lingual Gyrus	R	10110	12	–84	–8	6.54	0
Paracentral Lobule	R	1243	10	–44	64	6.06	0
Mid-Cingulum	L	126	0	–14	40	3.83	0.01

*Peak coordinates for main clusters are reported in Montreal Neurological Institute (MNI) space (x, y, z).*

#### Region-of-Interest (ROI) Analysis

##### Independent ROI analysis

To examine changes in the neural basis of risk representation in the medial prefrontal region, we conducted independent ROI analyses based on separate masks taken from a previous study we performed that found risk-related signals in the ACC (peak voxel = MNI 6, 26, 24) and bilateral IFG /anterior insula (IFG/AI) (peak voxels = MNI –44, 16, –8 and 48, 20, –6) (Fukunaga et al., 2012). That study used the Balloon Analog Risk Task (BART) and identified areas where BOLD activation related to choosing to gamble increased as the probability of explosion increased. As we had access to the full ROI mask, we used all voxels in each mask, not just the peak voxel. We tested for the significance of the variance regressor in these *a priori* regions and found significant variance loading in the ACC [*t*(24) = 1.796, *p* = 0.0425, one-tailed] and right insula [*t*(24) = 3.599, *p* < 0.001, one-tailed], but not in the left insula [*t*(24) = 0.234, *p* = 0.4083, one-tailed]. We note that our tests here are one-tailed because of the prior hypothesis of increased activation with greater risk, and the result in the ACC would not survive a two-tailed test, although the result in the right insula would survive.

### Relationship Between Neural Activity and Risk Aversion

If ACC and the MFG/IFG region activities that show effects of gamble variance are related to risk avoidance, then we would expect greater neural activity loading on the *Var* regressor to be associated with smaller average CE s, thus reflecting greater risk aversion. To test this, we calculated for each participant the average activation for when a gamble option was chosen for each of the five gambles, in the ACC region identified above (MNI 16, 32, 44). An ANCOVA with the five gambles as a fixed factor was performed in order to evaluate whether CE served as a covariate to ACC activation. Results were non-significant [*F*(1,95) = 1.23, MSE = 0.207, *p* = 0.27]. We also used a small-volume correction consisting of the region of ACC and overlying pre-SMA defined as loading on the variance regressor (*cf.* **Figure 3**) to investigate a potential correlation between the variance activation in the ACC (MNI 16, 32, 44) and average CE across subjects, but there was still no significant effect. Furthermore, there was no significant correlation between average CE across subjects and the GLM choice regressors that were parametrically modulated by loss probability or maximum possible loss.

We further explored whether ACC and MFG/IFG gamble variance effects might correlate with CE only when the subject chose the gamble or the ST option. To do this, we constructed a new set of GLMs in which trials when the subject chose the gamble were modeled separately from those in which subjects chose the ST. First, a test of main effects showed greater activity when subjects chose the Gamble vs. ST in the bilateral dorsal striatum (MNI -10, 0, -14), consistent with reward seeking, as well as in the precuneus and middle frontal gyrus (**Supplementary Table S8**). The opposite contrast showed significant effects in the superior frontal gyrus and cerebellar culmen (**Supplementary Table S8**).

We found regions with both positive and negative correlations of gamble variance on CE across subjects (**Supplementary Table S8**), including a positive correlation in the bilateral posterior insula (MNI 42, -12, -8 and MNI -38, -22, 2) between CE and the magnitude of the response to gamble variance, specifically when subjects chose the gamble. We also found a negative correlation in the insula (MNI 38, -16, -8), consistent with a role for the insula in risk aversion. The remaining gamble variance correlation effects with CE were found in posterior regions (**Supplementary Table S8**). Regarding other effects, we also found a significant correlation across subjects between the magnitude of the variance-related activation in the ACC and the DOSPERT expected benefits scale (**Supplementary Materials, Supplementary Figure S5**).

## Discussion

### Task Performance

In this study, we have aimed to adjudicate among competing definitions of risk, and we find consistent evidence in favor of variance as a formal definition of risk most closely related to how the brain represents risk. We evaluated neural correlates of risky decision-making using a novel gambling task that orthogonalized each gamble’s variance, loss probability, and maximum possible loss, while maintaining a constant expected value. Behavioral findings indicated that greater variance was associated with lower CEs, an indicator of risk avoidance, and counterintuitive to expectation, P(*Loss*) was positively correlated with CE. This is surprising considering previous behavioral findings suggest that P(*Loss*) is perceived to be riskier than both *Var* and Max(*Loss*), and impacts the perception of variance across financial gambles (Duxbury and Summers, 2004).

### Risk-Related Neural Activity

It has been posited that risk aversion to mixed gamble options is a result of enhanced sensitivity to value and loss aversion, but that risk is simply a factor in overall option value representation, and not a distinct representation of risk or potential loss (Tom et al., 2007). In contrast, ACC and insula/IFG activity has been shown to correlate more specifically with risk and avoidance in previous literature (Brown and Braver, 2007; Preuschoff et al., 2008; Krawitz et al., 2010; Congdon et al., 2013). ACC is considered to be a part of an integral network involved in decision-making and evaluation, but its role is contested, with numerous proposed functions (Ebitz and Hayden, 2016). Our main findings suggest that ACC activity may signal among other things a particular kind of representation involved in decision-making, namely the variance of the potential outcomes. Specifically, the variance of a gamble payout distribution may be a better formalism by which to model risk at the neural level, as opposed to other constructs such as loss probability or maximum possible loss (Brown and Braver, 2005, 2007). Our results provide perspective on earlier work showing that insula activation was associated with reducing the possible loss (Venkatraman, et al., 2009), but in that study, reducing the loss necessarily also reduced the variance, such that loss minimization and variance minimization were confounded. Here we disentangle the potentially confounded factors and show that specifically variance minimization is associated with ACC and insula activation. Furthermore, increased variance-related activation of the ACC was shown to correlate with decreases in the expected benefits of risky behavior (**Supplementary Figure S5**). Computationally, it appears that the brain is able to at least represent variance without necessarily representing maximum possible loss or loss probability *per se*. This is not surprising, as the calculations are different, and variance can in principle be computed without identifying the probability of loss specifically or the maximum possible loss.

### Competing Definitions of Risk

Our results shed light on continuing disparities in the literature regarding the definition of risk. Our finding of increased ACC activation with increasing gamble payout variance is consistent with the concept of risk as defined in literature in economics (Markowitz, 1952) and neuroscience (Preuschoff et al., 2006). Our findings are not as consistent with definitions of risk as the probability of a loss *per se* (Saaty, 1987; Rothman and Salovey, 1997; Duxbury and Summers, 2004), at least at the level of neural representation. While the precise roles of the ACC and AI/IFG in decision-making and risk evaluation remain contested, we provide further support for their role in risk evaluation and decision-making (Amiez et al., 2016).

### Relationship Between Neural Activity and Behavior

It is noteworthy that we found no correlation between variance regressor loading in the ACC and the CE s across participants. Based on previous work (Brown and Braver, 2007; Fukunaga et al., 2012), we expected but did not find greater risk-related activity in the ACC to correlate with reduced valuation of the gamble and thus lower CEs. One possible reconciliation of this discrepancy is the idea that ACC variance signals may drive a devaluation of the reward associated with an option rather than avoidance of risk *per se*, consistent with our findings of reduced expected benefits with greater variance-related ACC activity. Previous studies found that greater ACC activation was associated with greater expected loss associated with a reduction in the expected value of the trial (Krawitz et al., 2010). Here, we controlled for expected value by holding it constant across all five gambles. Our design could thus detect signals associated with risk aversion (i.e., aversion to higher variance gambles), but it could not detect signals associated with reduced value. Nevertheless, we cannot strictly rule out the possibility that some brain regions may represent loss probability or maximum possible loss solely on the basis of the current null results.

Another possible account of ACC activation is that it may increase when a decision is made to engage in a suboptimal choice, whether that involves too much risk or too little risk (Hewig et al., 2009). As an aside, we attempted to assess this directly in the present data set with an additional GLM analysis that modeled the choice of Gamble and “ST” option separately at the time of choice, for each of the five gambles individually, and with an additional regressor for each condition that was parametrically modulated by the value of the ST. We reasoned that if ACC signaled an impending suboptimal choice, then the choice of a gamble option should lead to greater activation with increasing values of the ST, and likewise the choice of a ST option should lead to greater activation with decreasing values of the ST. We found no evidence for or against either of these hypotheses, which we did not consider surprising given the fact that the values of the ST option were intentionally clustered around the CE value for each gamble. As a result, this left little variation in the ST values that could otherwise be exploited to identify correlated neural activity. Thus our data are not well suited to answer this follow-up question.

Recent work suggests several alternative theories of ACC function. One theory suggests that ACC signals the value of foraging (Kolling et al., 2012, 2016). While ours is not specifically a foraging task, we analyzed the trials in which subjects chose the gamble over the ST option. Gambles with larger variances could be conceptualized as exemplifying foraging behavior, as there is more certainty with smaller variances (Weber et al., 2004), as such our results could inform studies of foraging. We would expect ACC activity to be larger when subjects choose a gamble with a larger variance (analogous to the greater uncertainty associated with a decision to forage), which is precisely what we found in our study. Similarly, we found that subjects with greater ACC activity generally perceived a lower level of expected benefits of certain activities as measured by the DOSPERT (See **Supplementary Figure S5**). This is consistent with a drive to forage when the expected benefits of available resources are low. Another theory suggests that ACC represents the expected value of control (Shenhav et al., 2013). In our study, we controlled for expected values, which meant that there was no greater expected value of gain by virtue of choosing a gamble with a greater variance. Based on this critical difference in experimental design, our results are not obviously consistent with an interpretation in terms of the expected value of control, but neither do they rule out such an effect that may not have been detectable given our experimental design.

Another more recent theory suggests that rather than signaling foraging value, the ACC signals the difficulty of a decision (Shenhav et al., 2014). Here again, it is not clear that the ACC loading on the variance regressor is consistent with this notion, because the choices on both higher and lower variance gambles were uniformly difficult – participants chose the gamble option approximately 50% of the time for each of the gambles due to the staircase algorithm that adjusted the CEs to maintain the 50% choice probability. What we can conclude based on our study design is that the ACC signals the variance of a chosen gamble, which may reflect a drive to forage, and more indirectly, to avoid loss. We did not find more direct evidence of control effects here, a point which we return to below.

Regarding the origin of the variance signal, our results are consistent with the PRO model of ACC (Alexander and Brown, 2011, 2014). In the PRO model, individual cells predict each possible outcome of an action, and each prediction is compared against the outcome to generate a prediction error. The present results suggest that, in the framework of the PRO model, prediction-related ACC cells may have a kind of receptive field. Thus a given cell would be maximally activated by a particular possible outcome value (the preferred value of the cell), and the activation would be weaker as possible outcomes differ from the cell’s preferred value. A wider range of possible outcomes may lead to activation of more distinct populations of prediction cells, leading to greater overall summed activation across the population of cells. Conversely, lower variance gambles may lead to overlapping activation of the same cell populations to represent the various possible outcomes, leading to a smaller set of active cells and hence reduced activation when summed across the population. If so, then that implies a prediction for empirical investigation, namely that individual prediction-related cells in ACC should show receptive fields for particular predicted outcomes.

### Limitations

There are several main limitations to our findings. First, while our results shed light on the nature of ACC activity at the time of decision-making, it is less clear from our results what is the direct effect of that ACC activation on control of behavior, as our experiment was not designed to directly address this question. Second, the gamble outcome distributions were designed to orthogonalize the loss probability, maximum possible loss, and variance while maintaining expected value constant. The tradeoff for doing so meant that the maximum possible *win* was almost perfectly correlated with the variance of the gambles (*r* = 0.998), which opens us to the possibility that the regions sensitive to gamble variance may be sensitive to the maximum possible win value in addition to or instead of the variance. Nevertheless, such reward sensitivity is less likely given previously reported results, in which ventromedial prefrontal cortex rather than dorsal ACC has been shown to be sensitive to the reward value of the chosen option (McClure et al., 2004; Plassmann et al., 2008; Fukunaga et al., 2012). In contrast, ACC activity has instead shown to signal potential losses by increasing its activity when less valuable options are chosen (Fukunaga et al., 2012), especially when individuals more often avoid the less valuable options (Krawitz et al., 2010). Previous literature thus strongly suggests that ACC activity is specifically associated with avoiding less valuable options, which is also consistent with anatomical studies showing a preponderance of inhibitory output signals from the ACC to lateral prefrontal cortex (Medalla and Barbas, 2009). A related third limitation is that specifically the fifth gamble has the largest variance of all the gambles, so we cannot rule out the possibility that the greater ACC activity associated with gamble variance is driven strongly by another to-be-determined property of the fifth gamble specifically (e.g., treatment of the lowest-probability possible outcome; Kunreuther et al., 2001). Still, even if this were the case, our results stand in that factors at least related to the gamble variance more potently elicit ACC activity than factors related to loss probability or maximum possible loss.

As an aside, we also considered *post hoc* analyses of whether the gamble properties might correlate with other formal metrics, especially entropy and skewness of the gambles. We found that the entropy of the gambles shared 92.4% of the variance with loss probability. The skewness of the gambles shared over 99.7% of the variance with the gamble variance. These correlations depend only on the structure of the gambles, not on subject behavior. Given that, we concluded that loss probability and variance were likely to reflect the properties of entropy and skewness, respectively, and so we did not consider them further here. It should be noted that given the strong shared variance between skewness and gamble variance, we cannot rule out that the observed effects of gamble variance might be attributed to skewness as well.

The fourth limitation lies in the fact that having five gambles that are repeatedly observed may create a non-independence issue, and specifically in that treating the five gambles as a fixed effect rather than a random effect may limit the generalizability of the findings to other kinds of gambles. This limitation is common to nearly all existing fMRI studies which study a finite set of experimental conditions and has only recently been pointed out (Westfall et al., 2016). This potential limitation could be remedied by random rather than fixed effects modeling, but random effects for experimental factors are not yet supported in typical fMRI analysis packages such as AFNI, FSL, SPM (Westfall et al., 2016). Not accounting for mixed-effects may result in marginal alpha inflation for fixed, relative to random, effects, and thus our results can best be understood as inferences on the formal properties of risk in the specific gambles we studied.

Fifth, the task design, while targeted toward orthogonalizing risk formalisms, creates several limitations which are important to acknowledge. As ST values were titrated such that subjects chose the gamble in 50% of gambles, it is possible that the lack of findings in P(*Loss*) and Max(*Loss*) were influenced by a lack of motivation as a consequence of subjects’ risk evaluation being near the indifference point.

A sixth limitation may the relatively small sample, and that most of our subjects were risk seeking. Indeed, in separate analyses that considered potential systematic nonlinearities in probability and value perception (not detailed here), all but two of our subjects had best-fit value function exponents greater than 1 when we fit them to Prospect Theory (Paulus and Frank, 2006), consistent with risk-seeking, and precluding analysis of only risk averse subjects. Nevertheless, our results highlight that within a given subject, higher variance was associated with lower CEs, and not with higher CEs as would otherwise be expected with risk-seeking subjects. This indicates, incidentally, that systematic nonlinearities in value and probability perception may not fully account for subject risk preferences here. Our argument and conclusions are predicated on the relative preference of difference variance levels *within* subjects, and not on whether the subjects were on average risk seeking or risk averse.

More generally, it has been said that all models are wrong, but some are useful (Box, 1976). We show here that certain brain regions are sensitive to the variance of a gamble, and that the gamble variance is negatively correlated with preference, but we hesitate to claim that the brain computes variance in the mathematical sense. We do not know exactly what the brain computes, only that it is sensitive to variance and not to related formal constructs such as loss probability or maximum possible loss when the expected value of differing gambles is constant. In particular, we cannot rule out that some yet-to-be-determined idiosyncratic property of our gambles elicits a particular preference or aversion. Indeed, even with many different gambles we cannot rule out that the brain will have a particular preference for a particular gamble, as there are potentially infinitely many parameters that could fit (or overfit) the neural response to particular gambles. Given that, our question is more circumscribed: given that all models are wrong, which is more useful? The answer given by our data is that the gamble variance is a more useful construct.

While our results show effects of risky decisions involving money, it remains unclear whether our findings will generalize to other domains and contexts such as social or recreational risk taking (Figner and Weber, 2011; Weber et al., 2002). For our study, we selected risk parameters of monetary loss and gain. These are ideal because they can be well controlled in an experimental setting; however, we recognize that decision-making behaviors within a single risk domain may not generalize (Gilman et al., 2015), or be explained by existing economic theories (e.g., CE, risk-taking; Kusev et al., 2009). While this study focuses specifically on financial risk taking, future work might assess risk-taking behaviors across domains in order to understand how risk preferences correspond with task performance (Finucane et al., 2000) and neurological correlates (Rudorf et al., 2012).

## Conclusion

Our study findings directly address the question of how ACC activity may provide a basis for cognitive control of behavior. This study provides a unique design not applied elsewhere, such that we intentionally controlled for the ST alternatives so that from the participant’s perspective, neither the gamble option nor the ST option were more or less subjectively valuable than the alternative. If ACC provides a control signal to bias against less valuable options, it could be argued that there is no subjectively less valuable option to bias against based on this study design. Previous studies arguing for a role of ACC in risk avoidance did have an objectively less valuable option (Fukunaga et al., 2012), which was also subjectively less valuable as indicated by lower choice probabilities (Krawitz et al., 2010). In the case of choosing between options with differing subjective values, ACC may well drive loss avoidance as a bias against the less valuable option. Specifically, where the gamble is less valuable, ACC activity may bias against the gamble, hence a risk-avoidance effect as previously observed (Krawitz et al., 2010; Fukunaga et al., 2012). Where instead the riskier option is *more* valuable, the ACC may bias against the safe option, in favor of riskier options as in foraging (Hewig et al., 2009; Kolling et al., 2012, 2016).

In sum, we conclude that our results are consistent with the notion of ACC activity guiding decisions toward more valuable options and against less valuable options. Whether a riskier (higher variance) option is favored or not may depend more on whether it is more valuable, regardless of whether it is risker or not (Walton et al., 2002; Shenhav et al., 2014), thus making choices more rational (Paulus and Frank, 2006). In the end, the ACC may reflect a prediction of all possible outcomes, which serves as a basis for controlling behavior to avoid less valuable options, whether those options involve more or less variance. Current computational neural modeling work on ACC is consistent with these notions (Alexander and Brown, 2010; Brown and Alexander, 2017). There may also be other facets of risk that we have not examined here.

## Ethics Statement

This study was carried out in accordance with the recommendations of the Indiana University Institutional Review Board. The protocol was approved by the Indiana University Institutional Review Board. All subjects gave written informed consent in accordance with the Declaration of Helsinki.

## Author Contributions

JB and RF designed the experiments. RF collected the data. RF, JP, and JB analyzed the data and wrote the paper.

## Conflict of Interest Statement

The authors declare that the research was conducted in the absence of any commercial or financial relationships that could be construed as a potential conflict of interest.

## Footnotes

1 http://afni.nimh.nih.gov/afni

2 www.fil.ion.ucl.ac.uk/spm
