# Supplementary material for: Discriminating Formal Representations of Risk in Anterior Cingulate Cortex and Inferior Frontal Gyrus
Source: Front Neurosci. 2018 Aug 14;12:553. doi: 10.3389/fnins.2018.00553 (PMC6102448; doi:10.3389/fnins.2018.00553)
Supplement: Supplementary file 1 [file Data_Sheet_1.pdf]

Title: Discriminating formal representations of risk in anterior cingulate cortex and inferior frontal gyrus

Authors: Rena Fukunaga, John R. Purcell, and Joshua W. Brown

# Supplementary Methods

**Table S1. Specifics of Gamble Offers including gain and loss value magnitudes and probability of occurrence.**

| Gamble 1 | | Gamble 2 | | Gamble 3 | | Gamble 4 | | Gamble 5 | |
|---|---|---|---|---|---|---|---|---|---|
| Value | Prob | Value | Prob | Value | Prob | Value | Prob | Value | Prob |
| 30 | 0.8 | 30 | 0.8 | 30 | 0.5 | 30 | 0.5 | 0 | 0.82 |
| -30 | 0.1 | -65 | 0.1 | -8 | 0.25 | -65 | 0.25 | -23 | 0.13 |
| 90 | 0.1 | 125 | 0.1 | 68 | 0.25 | 125 | 0.25 | 660 | 0.05 |

**Table S2. Pearson correlations across the formal properties of the five gambles and sure thing**

| | **P(Loss)** | **Max Loss** | **Entropy** | **Skewness** |
|---|---|---|---|---|
| **Variance** | 0.0026 | -0.0050 | -0.0455 | 0.9987 |
| **P(Loss)** | | -0.0373 | 0.9613 | -0.0316 |
| **Max Loss** | | | -0.2585 | 0.0308 |
| **Entropy** | | | | -0.0856 |

**Table S3. Differences in Certainty Equivalent (CE) for each gamble.** Gamble 1 = Baseline, Gamble 2 = Control Probability of Loss, Gamble 3 = Control Variance, Gamble 4 = Control Maximum Possible Loss, Gamble 5 = Orthogonalization Condition. For significance levels (2-tailed); * ≤ .05, ° ≤ .01, † ≤.001

| | Gamble 2 | Gamble 3 | Gamble 4 | Gamble 5 |
|---|---|---|---|---|
| Gamble 1 | ($t$(24)=3.03)° | ($t$(24)=-2.08)* | ($t$(24)=.88) | ($t$(24)=6.12)† |
| Gamble 2 | | ($t$(24)=-3.31)° | ($t$(24)=-0.87) | ($t$(24)=5.00)† |
| Gamble 3 | | | ($t$(24)=2.68)* | ($t$(24)=6.81)† |
| Gamble 4 | | | | ($t$(24)=5.56)† |

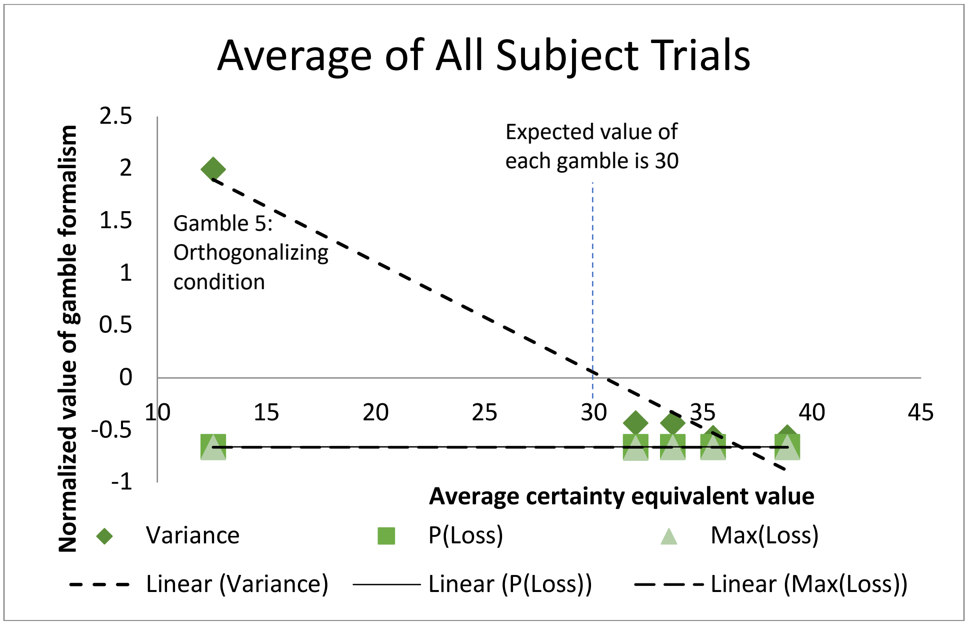


**Figure S1. Average plot of average certainty equivalent value for all subject trials separated by gamble formalism.** The expected value of each of the five gambles is 30, so certainty equivalents greater than 30 indicate risk seeking.

**Table S4. Certainty equivalents (CEs) for each subject and each gamble, with average CE per subject.**

| Average CE | Gamble 1 | Gamble 2 | Gamble 3 | Gamble 4 | Gamble 5 |
|---|---|---|---|---|---|
| 39.87 | 46.558 | 40.417 | 49.279 | 45.212 | 17.886 |
| 30.009 | 33.829 | 33.912 | 36.835 | 37.331 | 8.137 |
| 25.536 | 30.221 | 22.856 | 32.02 | 42.29 | 0.291 |
| 29.133 | 33.313 | 29.802 | 41.128 | 41.716 | -0.291 |
| 12.982 | 32.283 | 27.037 | 5.827 | 0.026 | -0.264 |
| 26.737 | 32.422 | 33.55 | 36.456 | 30.968 | 0.291 |
| 41.39 | 42.209 | 48.886 | 44.421 | 45.372 | 26.064 |
| 44.981 | 43.316 | 32.028 | 48.647 | 36.67 | 64.243 |
| 24.455 | 30.162 | 21.766 | 39.708 | 29.868 | 0.769 |
| 40.179 | 37.144 | 37.105 | 38.581 | 37.714 | 50.35 |
| 42.935 | 35.107 | 31.912 | 41.867 | 49.219 | 56.57 |
| 25.132 | 31.785 | 36.209 | 26.769 | 31.662 | -0.764 |
| 49.384 | 49.784 | 43.945 | 54.441 | 53.645 | 45.107 |
| 41.715 | 41.409 | 37.495 | 48.033 | 47.637 | 34.002 |
| 27.093 | 34.676 | 30.063 | 44.283 | 24.603 | 1.842 |
| 31.202 | 36.722 | 35.594 | 35.697 | 41.178 | 6.821 |
| 23.964 | 31.638 | 29.163 | 30.136 | 29.148 | -0.026 |
| 37.499 | 40.598 | 42.735 | 47.809 | 43.968 | 12.386 |
| 22.266 | 36.616 | 26.617 | 28.227 | 20.635 | -0.764 |
| -1.984 | -1.424 | -6.993 | 15.164 | -9.026 | -7.643 |
| 27.066 | 30.671 | 36.098 | 35.162 | 33.11 | 0.291 |
| 21.033 | 33.828 | 26.914 | 37.149 | 12.075 | -4.8 |
| 39.634 | 46.988 | 41.782 | 57.524 | 51.376 | 0.498 |
| 32.466 | 46.483 | 26 | 57.099 | 30.366 | 2.382 |
| 27.475 | 30.644 | 32.985 | 39.741 | 33.495 | 0.508 |

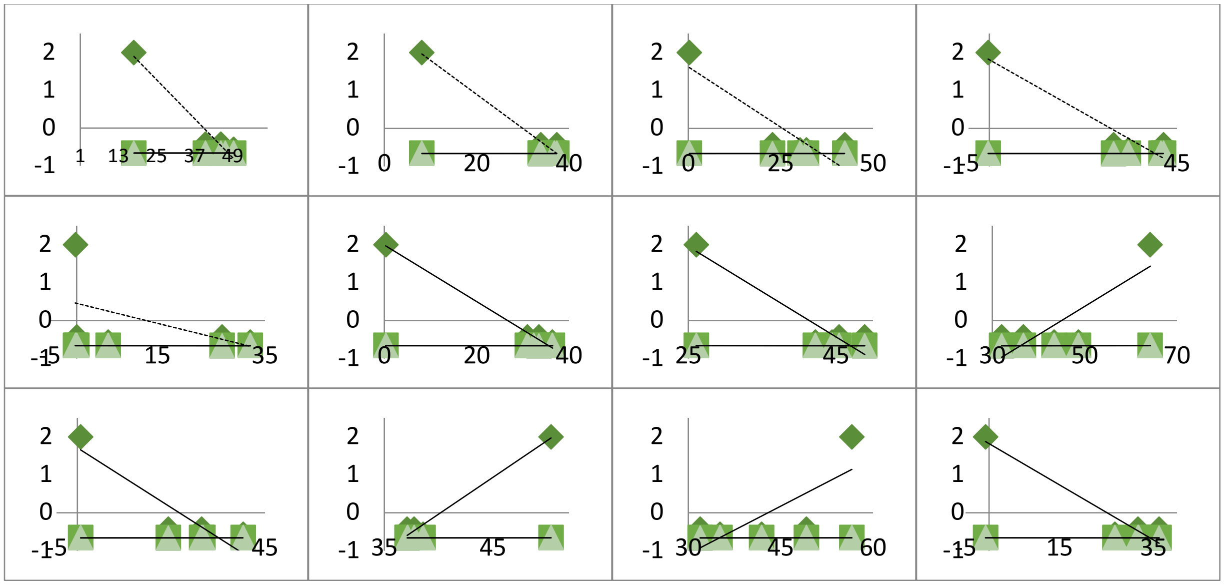

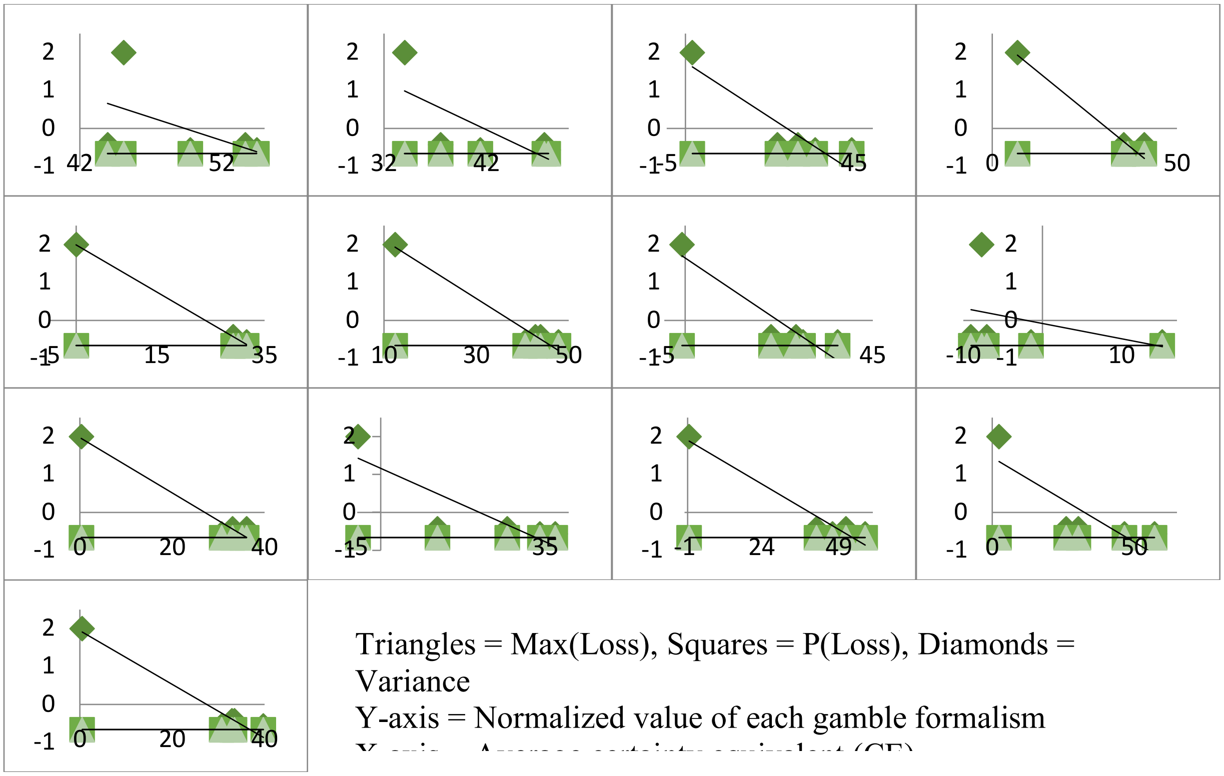


**Figure S2. Individual plots of average certainty equivalent value for each subject's trials separated by gamble formalism.**

**Table S5. Differences in log-transformed reaction times for each gamble**. Gamble 1 = Baseline, Gamble 2 = Control Probability of Loss, Gamble 3 = Control Variance, Gamble 4 = Control Maximum Possible Loss, Gamble 5 = Orthogonalization Condition. For significance levels (2-tailed); * ≤ .05, ° ≤ .01, † ≤.001

| | Gamble 2 | Gamble 3 | Gamble 4 | Gamble 5 |
|---|---|---|---|---|
| Gamble 1 | ($t(24)=-0.70$) | ($t(24)=2.53$)* | ($t(24)=-1.88$) | ($t(24)=4.22$)† |
| Gamble 2 | | ($t(24)=2.67$)* | ($t(24)=-1.43$) | ($t(24)=4.30$)† |
| Gamble 3 | | | ($t(24)=-3.40$)° | ($t(24)=1.94$) |
| Gamble 4 | | | | ($t(24)=4.95$)† |

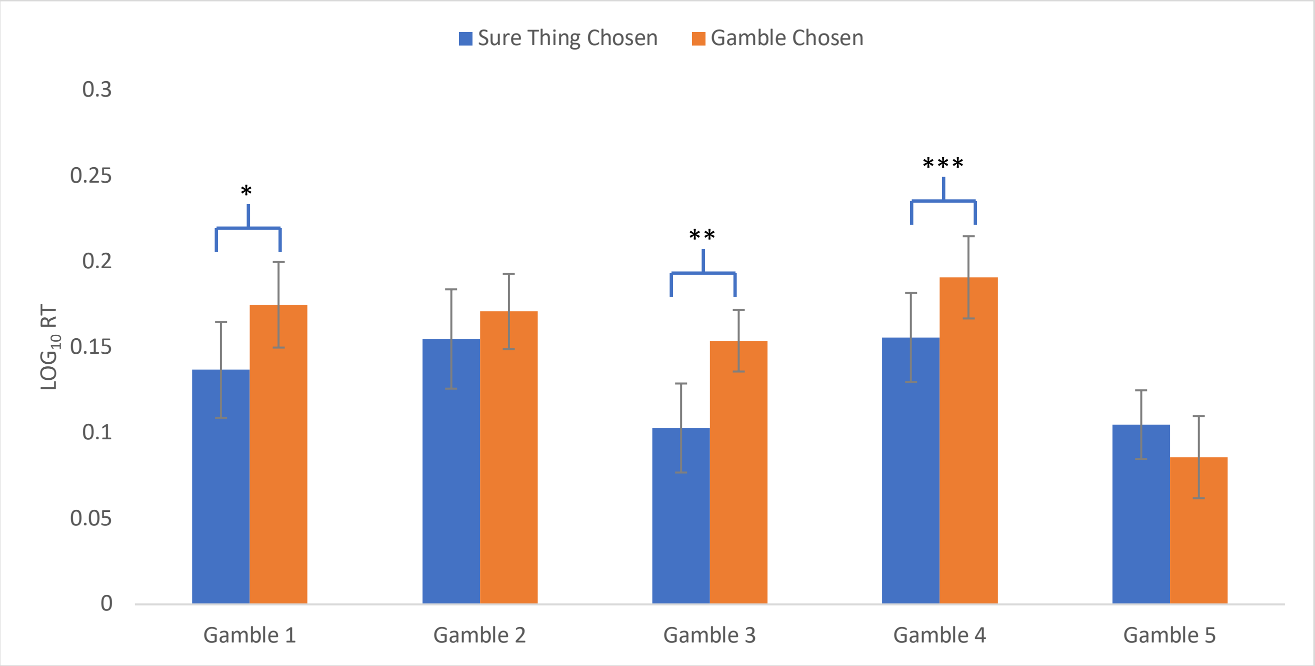


**Figure S3. Reaction Times for Trials Parsed by Gamble Chosen or Sure Thing Chosen on Each of the Five Different Gamble Parameters.** *Blue = $log_{10}$ transformed mean of all trials in which the sure thing (ST) option was chosen. Orange = $log_{10}$ transformed mean of all trials in which the gamble option was chosen. Gamble 1 = Baseline, Gamble 2 = Control Probability of Loss, Gamble 3 = Control Variance, Gamble 4 = Control Maximum Possible Loss, Gamble 5 = Orthogonalization Condition.* * = ($t(24)=-2.25$, $p = 0.034$), ** = ($t(24)=-3.49$, $p = 0.002$), *** = ($t(24)=-2.35$, $p = 0.027$).

Reaction times were compared between the average of trials in which participants chose the gamble option ($RT_{gamble}$) and the sure thing option ($RT_{ST}$). A paired-sample T-test revealed that the difference between $RT_{gamble}$ and $RT_{ST}$ approached, but did not reach, significance ($t(24)=-1.865$, p = 0.074).

The Pearson correlation between the Variance of the six options (five gambles and one sure thing) and the corresponding CE across the six options (noting that the variance of a sure thing option is zero). We then tested whether the Pearson correlation values across the population were significantly different from zero.

Within the trials in which the sure thing was chosen, there was a correlation between RT and MaxLoss ($t(24)=-2.515$, p = 0.019). Within the trials in which the gamble was chosen, there was a correlation between RT and MaxLoss ($t(24)=-2.649$, p = 0.014), and RT and Variance ($t(24)=-5.895$, p < 0.001). DOSPERT measures did not correlate with $RT_{gamble}$ or $RT_{ST}$.

**Table S6. Differences in log-transformed reaction times for gambles in which the sure thing was chosen.** Gamble 1 = Baseline, Gamble 2 = Control Probability of Loss, Gamble 3 = Control Variance, Gamble 4 = Control Maximum Possible Loss, Gamble 5 = Orthogonalization Condition. For significance levels (2-tailed); * ≤ .05, ° ≤ .01, † ≤.001

| | Gamble 2 | Gamble 3 | Gamble 4 | Gamble 5 |
|---|---|---|---|---|
| Gamble 1 | ($t(24)=-1.04$) | ($t(24)=2.13$)* | ($t(24)=-1.10$) | ($t(24)=1.61$) |
| Gamble 2 | | ($t(24)=2.70$)* | ($t(24)=-0.10$) | ($t(24)=2.08$)* |
| Gamble 3 | | | ($t(24)=-2.93$)° | ($t(24)=-0.10$) |
| Gamble 4 | | | | ($t(24)=2.52$)* |

**Table S7. Differences in log-transformed reaction times for gambles in which the gamble was chosen.** Gamble 1 = Baseline, Gamble 2 = Control Probability of Loss, Gamble 3 = Control Variance, Gamble 4 = Control Maximum Possible Loss, Gamble 5 = Orthogonalization Condition. For significance levels (2-tailed); * ≤ .05, ° ≤ .01, † ≤.001

| | Gamble 2 | Gamble 3 | Gamble 4 | Gamble 5 |
|---|---|---|---|---|
| Gamble 1 | (*t*(24)= 0.42) | (*t*(24)=1.77) | (*t*(24)=-1.32) | (*t*(24)=5.37)† |
| Gamble 2 | | (*t*(24)=1.45) | (*t*(24)=-2.00) | (*t*(24)=5.03)† |
| Gamble 3 | | | (*t*(24)=-3.03)° | (*t*(24)=3.57)° |
| Gamble 4 | | | | (*t*(24)=6.18)† |

**Table S8. Activation Differences During Task Feedback Presentation.** *Peak coordinates for main clusters (≥ 10 voxels) are reported in Montreal Neurological Institute (MNI) space (x, y, z). Results are Family-Wise Error (FWE) cluster corrected at P < .05*

| Region | Laterality | Cluster Size | Peak MNI Coordinates X | Y | Z | Max stat t |
|---|---|---|---|---|---|---|
| **Win Feedback – Loss Feedback Contrast** | | | | | | |
| Superior Frontal Gyrus | L | 1713 | -22 | -30 | 56 | 11.96 |
| Cerebral White Matter | R | 750 | 20 | -28 | 52 | 11.32 |
| Lingual/Occipital Fusiform Gyrus | R | 631 | 14 | -82 | -8 | 12.07 |
| Cuneus | R | 128 | 12 | -82 | 20 | 8.46 |
| Lateral Occipital | L | 116 | -16 | -88 | 18 | 8.20 |
| Precentral Gyrus | R | 53 | 6 | -18 | 52 | 7.62 |
| Orbitofrontal | L | 52 | -16 | 30 | -18 | 8.20 |
| Hippocampus | L | 27 | -28 | -28 | -14 | 8.37 |
| Cerebellar Lobules V/VI | L | 24 | -20 | -48 | -22 | 7.95 |
| Precuneous/Cerebral White Matter | L | 22 | -10 | -52 | 18 | 7.16 |
| Lingual Gyrus | R | 21 | 26 | -46 | -6 | 7.29 |
| Cerebral White Matter | L | 17 | -28 | -28 | 2 | 7.32 |
| **Loss Feedback – Win Feedback Contrast** | | | | | | |
| Paracingulate Gyrus | R | 286 | 6 | 42 | 28 | 9.17 |
| Frontal Orbital | R | 239 | 50 | 22 | -10 | 9.25 |
| Middle Frontal Gyrus | R | 196 | 36 | 18 | 28 | 8..85 |
| Superior Frontal Gyrus | R | 111 | 6 | 16 | 60 | 9.70 |
| Orbitofrontal/Insula | L | 77 | -36 | 18 | -12 | 8.79 |
| Cingulate | R/L | 52 | 0 | -18 | 26 | 7.79 |
| Midbrain | R | 36 | 4 | -30 | -4 | 7.76 |
| Temporal Occipital Fusiform Cortex | R | 27 | 44 | -50 | -22 | 7.84 |
| Temporal Occipital Fusiform Cortex | L | 12 | -44 | -50 | -24 | 7.53 |
| **ChooseGamble- ChooseSureThing Contrast** | | | | | | |

| | | | | | | |
|---|---|---|---|---|---|---|
| | | | | | | |
| Dorsal Striatum | L/R | 701 | -10 | 0 | -14 | 6.90 |
| Precuneus | L | 464 | -14 | -68 | 46 | 5.65 |
| Middle Frontal Gyrus | L | 162 | -38 | 32 | 38 | 5.14 |
| **ChooseSureThing-ChooseGamble Contrast** | | | | | | |
| Superior Frontal Gyrus | R | 148 | 24 | 46 | 22 | 5.74 |
| Cerebellum (Culmen) | L | 76 | -14 | -68 | -14 | 5.19 |
| **ChooseGamble*Var vs. CE (positive correlation)** | | | | | | |
| Insula (posterior) | R | 103 | 42 | -12 | -8 | 6.77 |
| Insula (posterior) | L | 109 | -38 | -22 | 2 | 4.20 |
| **ChooseGamble*Var vs. CE (negative correlation)** | | | | | | |
| | | | -- | | | |
| **ChooseSureThing*Var vs. CE (positive correlation)** | | | | | | |
| Parietal (angular gyrus) | L | 242 | -50 | -58 | 36 | 5.79 |
| **ChooseSureThing*Var vs. CE (negative correlation)** | | | | | | |
| Occipital | L | 324 | -38 | -74 | -6 | 5.92 |
| Insula | R | 303 | 38 | -16 | -8 | 5.41 |
| Occipital | R | 177 | 46 | -68 | -8 | 5.23 |
| Cerebellum (Culmen) | R | 208 | 28 | -48 | -28 | 4.94 |
| Parietal | R | 88 | 44 | -30 | 36 | 4.91 |

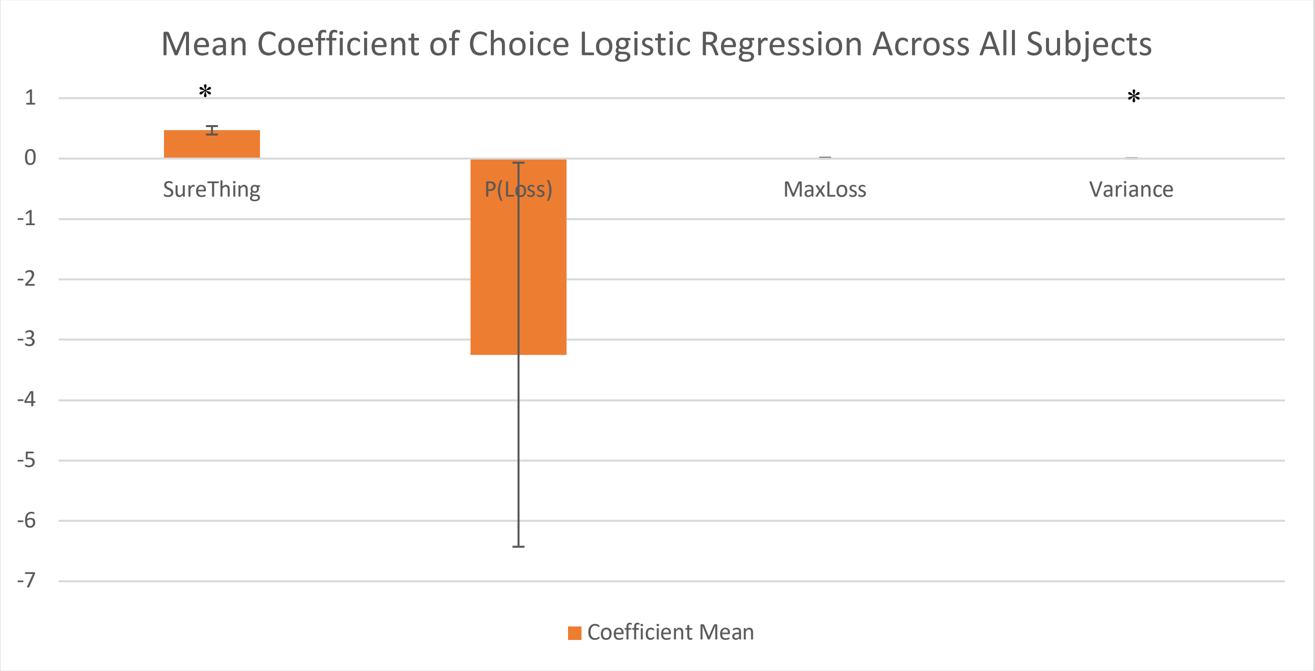


**Figure S4. Mean Coefficient of Choice Binary Logistic Across All Subjects.** In order to evaluate the choice of gamble vs. sure thing (ST) based on the available stimuli in that trial, a logistic regression was performed for each individual subject. Treating the binary of choice as the dependent variable, Sure Thing, P(Loss), MaxLoss, and Var were modeled as covariates. From here, independent samples t-tests across all subjects revealed significant results for ST ($t(24) = 5.935$, $p < .001$) and Var ($t(24) = 4.61$, $p < .001$), and non-significant for P(Loss) ($t(24) = -1.02$, $p = .32$) or MaxLoss ($t(24) = 0.86$, $p = -6.52$). Positive values signify risk aversion, whereas negative values signify risk seeking. The binary logistic regression equation was logit(P(Choose Sure Thing)) = $\beta_{\text{sure thing}}$*ST + $\beta_{\text{PLoss}}$*P(Loss) + $\beta_{\text{MaxLoss}}$*MaxLoss + $\beta_{\text{Variance}}$*Variance + $\beta_{\text{Constant}}$

| | **t(24)** | **P (2-tailed)** | **Mean value** |
|---|---|---|---|

| β | | | |
|---|---|---|---|
| SureThing | 5.935 | 0 | 0.46768 |
| PLoss | -1.02 | 0.318 | -3.24816 |
| MaxLoss | 0.868 | 0.394 | 0.00616 |
| Variance | 4.615 | 0 | 0.00184 |
| Constant | -6.517 | 0 | -15.5748 |

**Table S9. Population logistic regression coefficients for P(Choose Sure Thing)**

**No shift of gamble preferences over time**

It could be that the participants watched the same gambles repeatedly with the results of their certainty equivalents changing. To investigate whether their preferences changed with repeated gamble presentations, we looked at each of the five gambles, and we asked if the estimated certainty equivalent drifted over time. To do this, we looked for each subject and each of the five gambles to calculate the regression slope of the estimated certainty equivalent on trial number. For a given gamble, we asked whether the regression slope was significantly different across the population of subjects. We found that the slopes were not significantly different from zero for any of the five gambles (all $p > 0.05$).

**Verbal Instructions and Calculation of Monetary Task Bonus**

<u>Verbal Instructions:</u> In regard to the monetary task bonus, subjects were told, *"You will be compensated for your time, and you will also receive a bonus amount between $0 and up to $30. How much bonus money you receive will depend on your winnings. We will calculate your bonus amount by randomly selecting the outcomes of TWO of your decisions in the task. We will multiply the winnings by a number that we will tell you after the experiment has finished, and the resulting amount is the bonus amount that we will pay you. After the experiment has finished, we will pay you both for your time and for the bonus you won. The bonus amount we pay you will never be less than $0, so you will at least be paid for your time. Still, if one of the gambles that the computer chooses for your bonus payment is a loss, that will be deducted from any winnings in the other paid gamble."*

<u>Bonus calculation:</u> All bonuses were between zero and $30.00. Two of the task blocks were randomly chosen. Within these blocks a random "PaidTrial" was indicated in the data output. These are the raw winnings, Win1 and Win2. To compute the bonus amount, the following formula was used: Bonus = (Win1 + Win2)/48.33 + $2.69

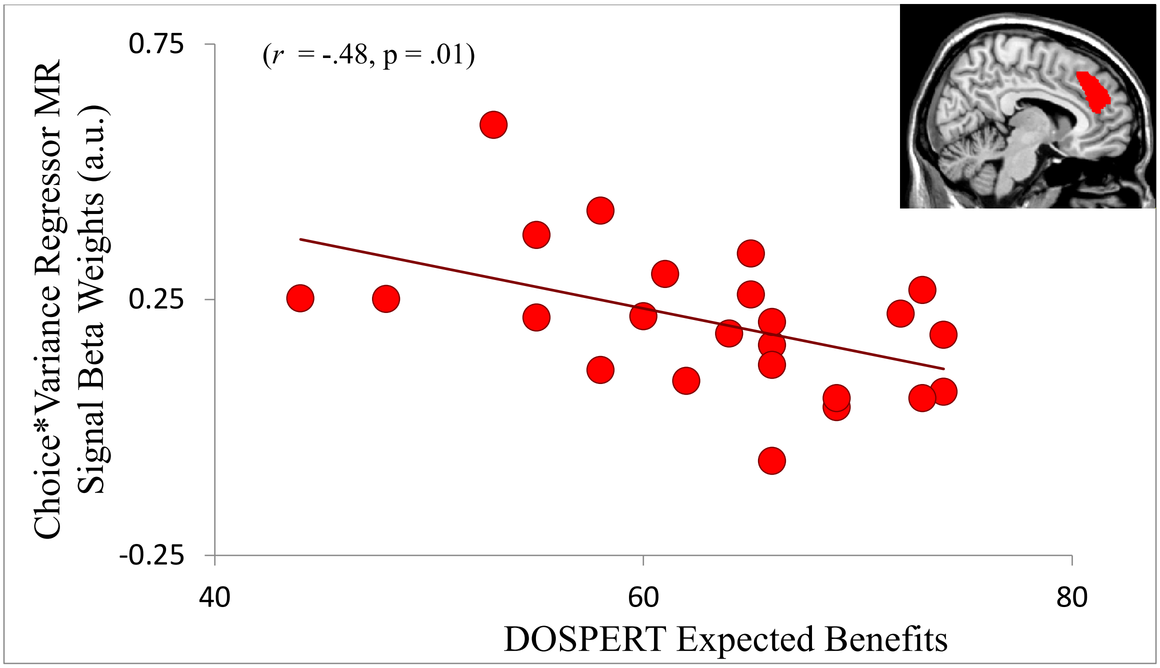


**Figure S5. Relationship Between Neural Activity During Choice and Behavior**. ROI defined independently as loading on the main effect regressor of variance, as shown in **Figure 3**. Greater risk-as-variance signals in ACC correlate with reduced DOSPERT expected benefits of risk-taking scores, which is consistent with a role in risk avoidance signals via reductions in expected benefits of risk-taking.

**Relationship between neural activity and self-report measures**

Despite the absence of a clear relationship between ACC gamble variance loading and CEs, we conducted *post-hoc* tests to examine associations between trait risk-taking and variance loading for the gamble condition in the ACC. Specifically, we hypothesized that greater risk-related activation should correlate with reduced expected benefits, as risk avoidance signals should lead subjects away from engaging in the risky behavior. Neural activation in the ACC (MNI 16, 32, 44) identified by the variance regressor was negatively correlated with self-report ratings of Total Expected Benefit of Risk-Taking ($r(22) = -.48$, $p = .010$, one-tailed) on the DOSPERT (Barkley-Levenson et al., 2013; Weber et al., 2002). We note that this is significant even when correcting for multiple comparisons of the correlation of ACC variance-related activity with the DOSPERT risk-taking likelihood and risk perception scales (Bonferroni corrected $\alpha = 0.016$). Further investigation identified that this effect was driven by the specific financial subscales of Investment Risk ($r(22) = -.53$, $p = .007$) and Gambling Risk ($r(22) = -.54$, $p = .006$), rather than the subscales of Recreational Risk ($r(22) =-.23$, $p = .27$) or Social Risk ($r(22) = -.18$, $p = .40$). These findings suggest that this ACC ROI may contribute

to the (down) regulation of expected benefits of task-relevant risky decisions, namely investing and gambling (Figure S5).

Our variance regressor loading in the ACC was correlated with decreased reports of expected benefits of risk taking on the DOSPERT, which suggests that ACC activity may reduce risk taking indirectly by reducing the perceived benefits of a risky action. The role of perceived risk versus expected benefits of a choice has also been debated (Weber & Johnson, 2009). The ‘affect heuristic’ of decision-making (Finucane, Alhakami, Slovic, & Johnson, 2000) suggests that there is an inverse relationship between risk perception and expected benefits, regardless of risk-taking behavior. This is ironic given that in real world decisions such as business, greater risk is often positively rather than negatively correlated with expected benefits. Expected benefits have been found to be positively correlated with risk taking behavior, despite being negatively correlated with perceived risk (Weller, Ceschi, & Randolph, 2015). Our results are consistent with this body of literature.

Previous literature has suggested that expected benefit from risk-taking may vary across domains of risk (Markiewicz & Weber, 2013; Zhang, Zhang, & Shang, 2016). For example, an individual who regularly gambles at a casino expects greater benefit from monetary risk than a bungee jumper, who expects greater benefit from recreational risk (Hanoch, Johnson, & Wilke, 2006). Our correlational findings between the DOSPERT expected benefit gambling/investing subscales and ACC (MNI 16, 32, 44) activation to variance provide further support for notions of domain-specific risk, as our task utilized gambling and financial (i.e. monetary) risk, specifically.

Somewhat surprisingly, we did not find an inverse relationship between the self-reported likelihood of financial risk taking on the DOSPERT and the loading on the variance regressor in the ACC or IFG. This was despite the negative correlation of variance regressor loading with the expected benefits of financial risk-taking. One possible reason for this lack of correlation is that several questions on the financial subscales of the DOSPERT involve scenarios with which college students may not identify, such as betting on horses or investing in speculative stock. Thus, our subjects may have been sensitive in the abstract to the risks and benefits of these behaviors even though they were less likely to engage in them.

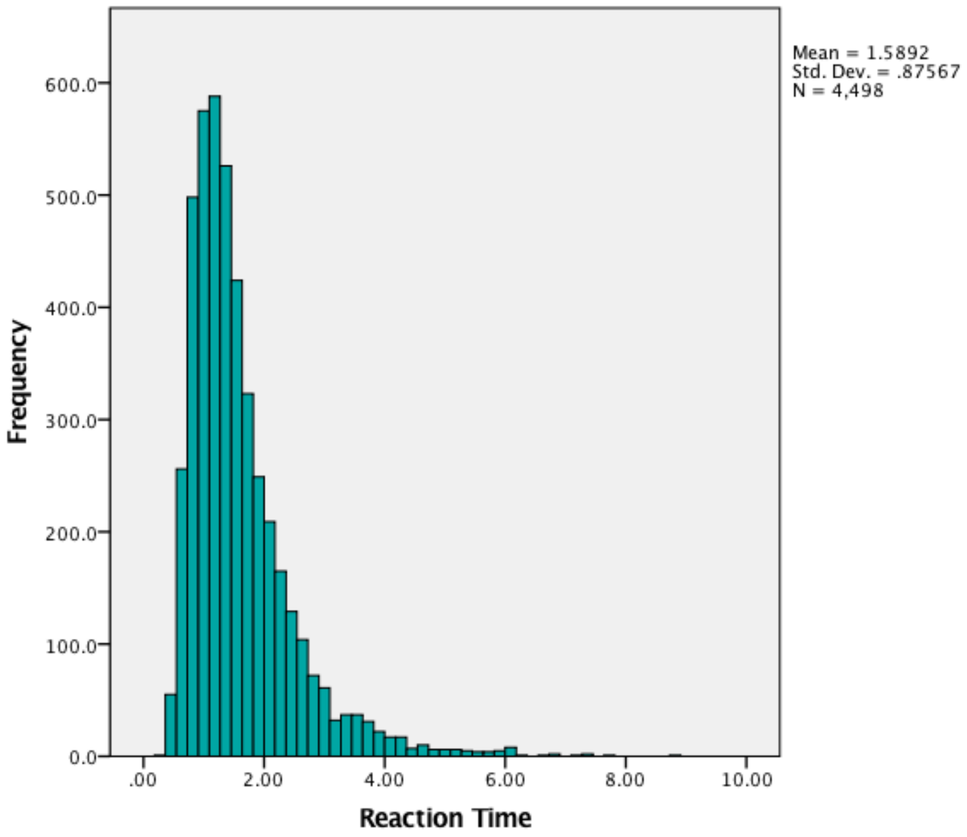


**Figure S6. Histogram of All Reaction Times in seconds.** Two data points not reported due to technical errors. RTs in the rest of the manuscript are $\log_{10}$ transformed prior to statistical tests to correct the non-normality.

## References


Barkley-Levenson, E. E., Van Leijenhorst, L., & Galván, A. (2013). Behavioral and neural correlates of loss aversion and risk avoidance in adolescents and adults. *Developmental Cognitive Neuroscience*, *3*, 72-83.

Finucane, M. L., Alhakami, A., Slovic, P., & Johnson, S. M. (2000). The affect heuristic in judgments of risks and benefits. *Journal of Behavioral Decision Making*, *13*(1), 1-17.

Hanoch, Y., Johnson, J. G., & Wilke, A. (2006). Domain specificity in experimental measures and participant recruitment: An application to risk-taking behavior. *Psychological Science*, *17*(4), 300-304.

Markiewicz, Ł., & Weber, E. U. (2013). DOSPERT's gambling risk-taking propensity scale predicts excessive stock trading. *Journal of Behavioral Finance*, *14*(1), 65-78.

Weber, E. U., Blais, A. R., & Betz, N. E. (2002). A domain-specific risk-attitude scale: Measuring risk perceptions and risk behaviors. *Journal of Behavioral Decision Making*, *15*(4), 263-290.

Weber, E. U., & Johnson, E. J. (2009). Decisions under uncertainty: Psychological, economic, and neuroeconomic explanations of risk preference. In *Neuroeconomics* (pp. 127-144).

Weller, J. A., Ceschi, A., & Randolph, C. (2015). Decision-making competence predicts domain-specific risk attitudes. *Frontiers in Psychology*, *6*, 540.

Zhang, L., Zhang, C., & Shang, L. (2016). Sensation-seeking and domain-specific risk-taking behavior among adolescents: Risk perceptions and expected benefits as mediators. *Personality and Individual Differences*, *101*, 299-305.
